# Supplementary material for: Carbon intensity of global crude oil trading and market policy implications
Source: Nat Commun. 2023 Sep 25;14:5975. doi: 10.1038/s41467-023-41701-z (PMC10520038; doi:10.1038/s41467-023-41701-z)
Supplement: Supplementary file 1 — Supplementary Information [file 41467_2023_41701_MOESM1_ESM.pdf]

Supplementary Information for

Carbon intensity of global crude oil trading and market policy implications

Yash Dixit[1], Hassan El-Houjeiri[2], Jean-Christophe Monfort[2], Liang Jing[3, 4], Yiqi Zhang[5], James Littlefield[4], Wennan Long[6], Christoph Falter[1], Alhassan Badahdah[2], Joule Bergerson[3], Raymond L. Speth[1*], Steven R. H. Barrett[1]

[1]Laboratory for Aviation and The Environment, Department of Aeronautics and Astronautics, MIT, Cambridge MA, USA

[2]Energy Traceability Technology, Technology Strategy and Planning Department, Aramco, Dhahran, Saudi Arabia

[3]Department of Chemical and Petroleum Engineering, University of Calgary, Calgary AB, Canada

[4]Climate and Sustainability Group, Aramco Research Center–Detroit, Aramco Americas, Novi, MI, USA

[5]Division of Environment and Sustainability, The Hong Kong University of Science and Technology, Hong Kong, China

[6]Energy Science & Engineering, Stanford University, Stanford CA, USA

*email: speth@mit.edu

Inventory of Supporting Information

Supplementary Note 1: Data sources – coverage and limitations 3
Supplementary Note 2: Summary of the methods 7
Supplementary Note 3: Edge construction in the network 7
Supplementary Note 4: Blend estimation algorithm 9
Supplementary Note 5: Tracking crude from oil fields to refineries 17
Supplementary Note 6: Estimation of upstream emissions 17
Supplementary Note 7: Estimation of midstream emissions 22
Supplementary Note 8: Literature references for midstream emissions 27
Supplementary Note 9: Contextualizing decarbonization based on crude CI differentiation 28
Supplementary Note 10: Relevant details on estimation of field-level upstream emissions 29
Supplementary Note 11: Data gaps and future research 31
Supplementary References 33

Supplementary Note 1: Data sources – coverage and limitations

We use commercial and publicly available high-fidelity data sources covering a variety of supply chain attributes – geospatial data (location of supply chain assets such as oil fields, shipping terminals, pipelines, refineries), crude trades data (market data mapping crude blends to refineries), shipping routes (timestamped locations of crude tankers), asset characteristics (properties such as production volumes of oil fields, specifications of pipelines) and miscellaneous supporting features (public datasets of ambient temperature, elevation)

Upstream – Crude oil production

The data used to map the upstream of the supply chain were obtained from commercial data sources and include the following features for global oil fields which represent ~98% of the global crude oil and condensate production in 2015 – geolocation and asset name, asset country, production volumes and properties of produced crude (density measured in API, sulfur content). This data was supplemented with data from Masnadi et al[1] which was used in this study to estimate the oil production carbon intensities using the latest, most advanced version of the Oil Production Greenhouse Gas Emissions Estimator (OPGEE version 3.0c)[2,3]. Sample oil fields in Norway with production volumes >50 k-barrels/day are shown in Supplementary Figure 1.

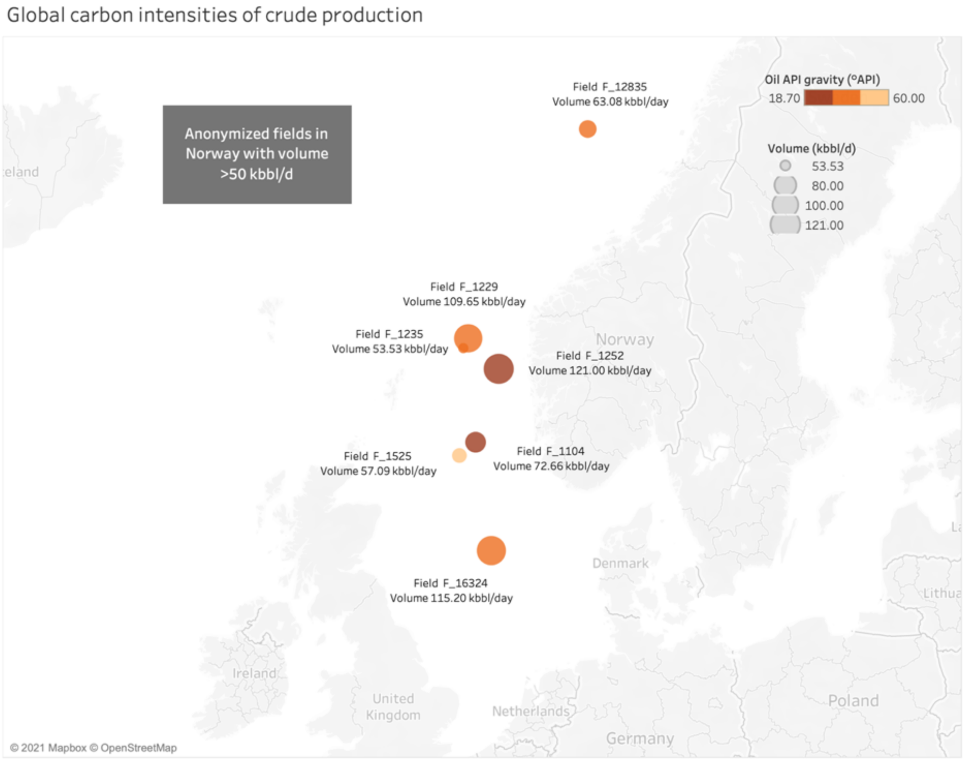

Supplementary Figure 1: Oil fields in Norway with production >50 k-barrels/day (kbbl/d). To illustrate the attributes of the upstream dataset, anonymized oil fields in Norway are shown

along with their volumes and crude APIs

Market trades

The market trades data which is also commercially provided has three key components – specifications of global marketed crude blends (volume, API gravity), demand data from refineries consuming the blends, geolocation data and asset identification of refineries. The specifications of global crude blends are key, particularly in conjunction with the upstream data. This feature is used to estimate how blends are formed from source oil fields as discussed in the Methods section of the main paper and later in Supplementary Note 4. We exclude NGLs and condensates from the analysis and hence attain ~95% coverage of 2015 global crude oil and condensate volumes. The downstream data i.e. mapping of crude blends to refineries is later used to create end-to-end traceability in the supply chain i.e. linking oil fields to refineries through information about crude blends. Sample refineries in India with locations and production volumes are shown in Supplementary Figure 2.

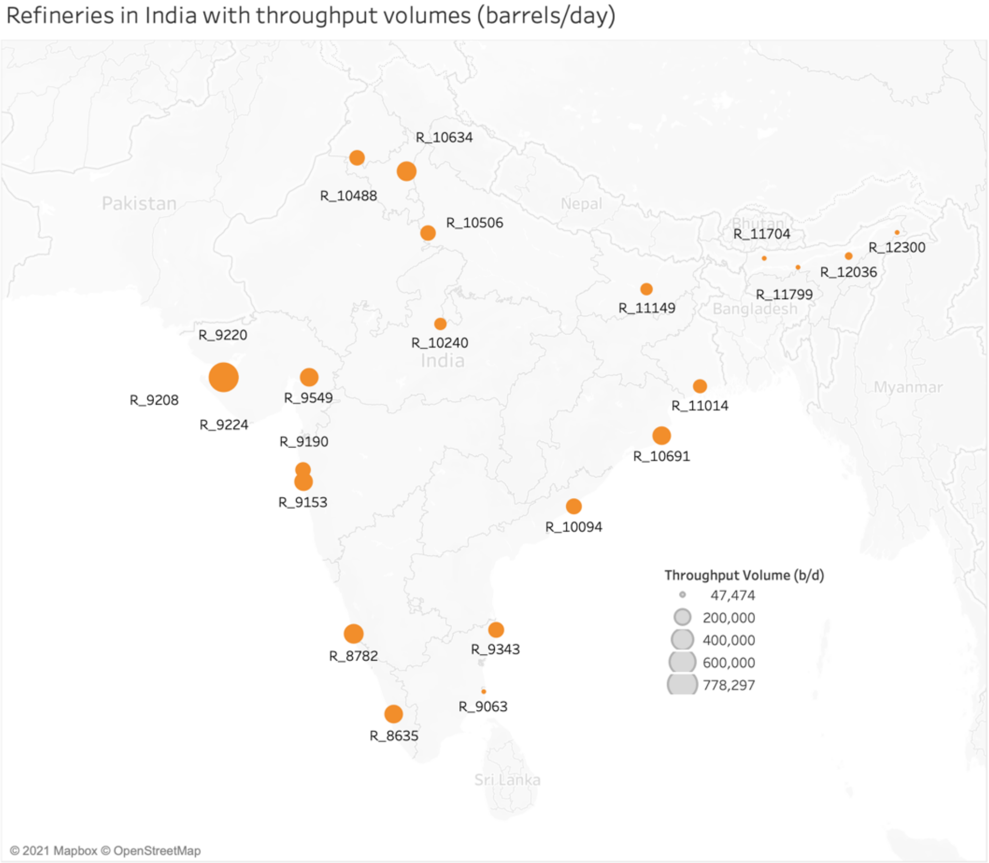

Supplementary Figure 2: Sample of the global refineries' dataset – with cumulative throughput volumes. Analogous to the upstream dataset, the downstream dataset entails the locations and consumption slates of refineries (specifically, the volumes and APIs of the crude

blends consumed by refineries). The figure shows locations and total throughput volumes for anonymized Indian refineries.

Supply chain infrastructure

Features pertaining to the rest of the supply chain data i.e. entities excluding oil fields and refineries are sourced from commercial providers GlobalData, Kpler and S&P Global[4–6]. The former two, put together, include locations of shipping terminals, locations of pipelines and asset characteristics of pipelines such as diameter, length etc. The latter provides crude tanker locations along with relevant features such as tanker engine configurations, speed, etc. which are necessary to estimate shipping emissions. High-fidelity estimates of these features at a granular level (e.g. individual pipeline segments, shipping routes) are of key importance in the bottom-up estimation of emissions associated with pipeline transportation.

These data sets are used to setup the network as described in Supplementary Notes 2 and 3. An example of how the locations of oil fields, refineries, and terminals along with the pipeline coordinates feed into the network is shown in Supplementary Figure 3.

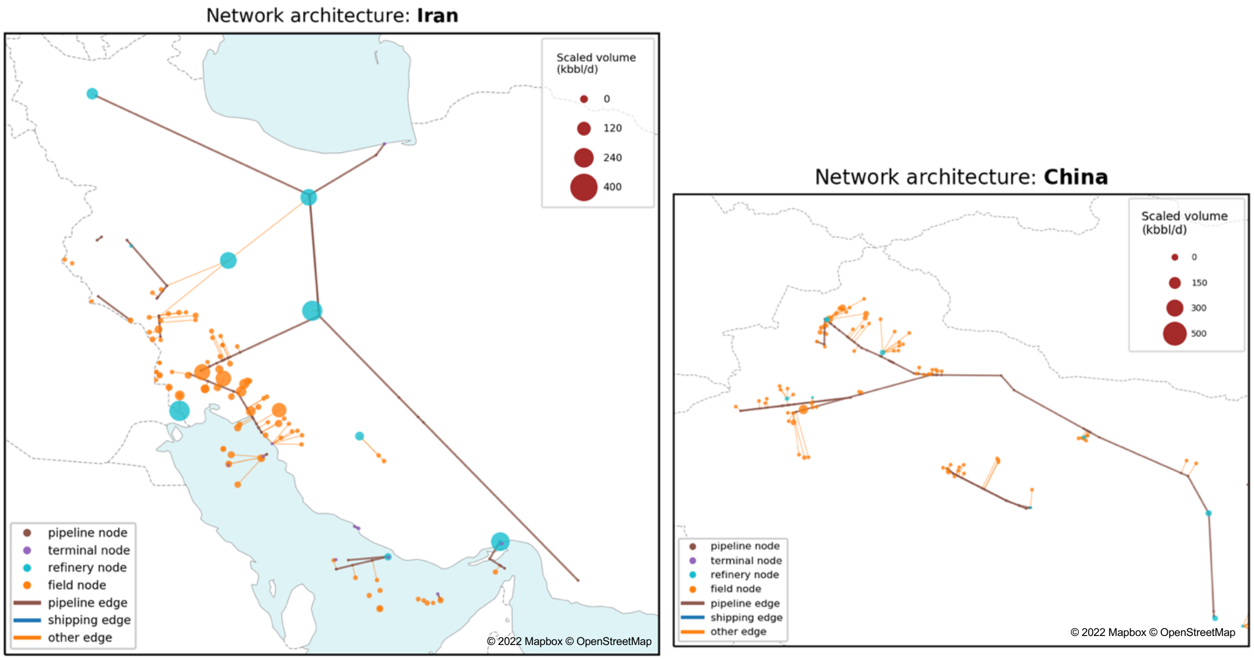

(a) (b)

Supplementary Figure 3: Sample of the supply chain network constructed from the oil fields, refineries, terminals, and pipeline datasets. The midstream datasets including, but not limited to pipeline and terminal locations are used in conjunction with the locations of oil fields and refineries. The global supply chain network is constructed using these data attributes as described in Supplementary Notes 2 and 3; figure (a) and (b) illustrate the network in Iran and Western China respectively.

The pipeline dataset provides high fidelity information such as precise geo-coordinates and diameter for pipelines across Europe, Middle East, Africa, Asia Pacific, Latin America and North America. Furthermore, the raw dataset has coverage of 6000+ oil and gas pipelines across 137 countries. Given the scope of the study on the "well-to-refinery entrance" emissions pertaining to the oil supply chain, we focus on the ~2500 crude oil pipelines from the dataset. As indicated in the main paper, the limited information about intra-field pipelines and the current representation of oil fields as point objects leads to the network needing other edges in addition to the pipelines from the raw dataset to be meaningfully connected. The broad coverage of the pipeline dataset is further substantiated by the fact that along non-shipping edges, the split of total barrel-miles in the network is 92% along pipeline edges (i.e. edges mapped to pipeline segments from the dataset) and 8% are along these "constructed" edges (refer to Supplementary Notes 3 and 4 for more details).

Geographical attributes

To construct physics-based emission models on top of the supply chain model, ancillary features such as ambient temperature and elevation are needed. For instance, elevation change across a pipeline is a pertinent input that guides emissions associated with pipeline transportation. For land and ocean temperature, we use the NASA MODIS Land/Ocean Surface Temperature and Emissivity data. The data is retrieved at 1 km pixels by the generalized split-window algorithm and at 6 km grids by the day/night algorithm[7]. In addition, elevation data is obtained at a resolution of 1 arc degree from two sources – the 2000 Shuttle Radar Topography Mission and NASA SRTM, which gives the elevation or altitude at any given geolocation[8].

Geographical coverage of the supply chain

The assessment includes 64 producer countries, 83 consumer countries with coverage across all populated continents (North America, South America, Africa, Europe, Asia and Oceania). In comparison with Masnadi et al. (Global carbon intensity of crude oil production)[1], which is the source for upstream field-level CI estimates used by our study, the following countries are excluded from the source side of the field to refinery tracking:

South Sudan, Niger, Netherlands, Afghanistan, Guatemala, Tajikistan, Pakistan, Cote d'Ivoire, Kyrgyzstan, Spain, Suriname, Jordan, Israel, Japan, Georgia, Bolivia, Greece, Morocco, Belize

This selection is done due to the absence of crude blends originating from the corresponding countries in the market trades dataset. This exclusion is salient given the assumption of the blend estimation algorithm which estimates blend formation from oil fields originating from the same country. These exclusions represent less than 0.5% of global field-level production volume. Note that, from these countries, all those which contain refining capacity are included in the destination side of the field to refinery tracking.

Supplementary Note 2: Summary of the methods

In continuation to the main paper, we present an overview along with the motivation for the key components of the methods:

Supplementary Table 1: Overview of the methods

Index	Goal of the method	Motivation	Overview of the approach
1	Modeling the supply chain	Efficient choice of data-structure for subsequent high-res carbon intensity estimation	Network-based approach - assets as nodes, transportation modes as edges
2	Estimating how oil fields combine to form marketed crude blends (Blend estimation algorithm)	Link sources (oil fields) and destinations (re- fineries) using shared info about crude blends	Multi-objective gradient-based optimization with an initialization algorithm
3	Tracking barrels from source oil fields to destination refineries	Generate visibility into how barrels move along supply chain pathways	Shortest-paths from oil fields to respective re-fineries based on crude blend data
4	Estimating carbon intensities along pathways	Quantify carbon emissions granularly and highlight decarbonization potential	Mode-specific emission estimation models (pipeline and shipping) + results from tracking

Supplementary Note 3: Edge construction in the network

Details about the three types of edges in the network (mode edges, concurrent edges and heuristic edges) are described below:

Mode edges

Mode edges are constructed using the datasets, specifically using pipeline geometries and shipping routes. These edges are assumed to be known precisely from data and are created between the corresponding nodes. In the case of a typical pipeline system which is defined in the dataset as a list of coordinates that correspond to nodes in the network, the edges are constructed between consecutive nodes to represent the system. The limitation of this chosen graphical

representation is that supply chain assets (oil fields, refineries, terminals) are assumed to be point entities, whereas in reality they are spatial entities. This limitation constrains the relationship between fields and pipelines in that, pipelines are typically observed to be constructed through the perimeter of oil fields which is hard to capture in the chosen representation. To manage this limitation, sub-segments of pipelines are created at junctions where field nodes intersect pipeline segments and thus allow fields to have proximate and efficient pipeline access. As a consequence, the network ends up having artificial pipeline junctions and a greater number of constituent pipeline segments. Relevant data features such as pipeline diameter and length are encoded as edge attributes. The other important mode edge captures shipping routes by linking shipping ports based on the dataset of export-import trades. Data features such as route mileage and tanker type are encoded as edge attributes. Mode edges represent the majority of the transportation pathways in the supply chain. The others enable a completion of the network by resolving issues of asset duplication, geolocation errors etc.

Concurrent edges

Concurrent edges are created between nodes which approximately have the same geolocation. A tolerance of 1 km is to construct edges which fulfil this criterion. This category of edges is salient in cases where asset classes like fields and refineries are key junctions in pipeline systems.

Heuristic edges

Heuristic edges are added to make the network representation more realistic. Partially aiming to address the aforementioned limitation of point entities, these edges are constructed between the field, refinery nodes and the rest. These heuristic edges use a thresholding criterion of 10 km and 50 km in a hierarchical manner i.e. if a particular field node lacks connectivity, it is connected to nodes within 10 km and if it still lacks connectivity the process is repeated with a 50 km threshold. These thresholds are chosen based on how well connected the network appears in terms of allowing for supply chain pathways from fields to destination refineries. More than 90 percent of the nodes are observed to secure connections after implementation of the three types of edges. The remaining are either:

a) Zero crude volume entities (defunct refineries, closed oil fields)
b) Terminals without any assets near them and without any shipping trades associated with them (likely down to misclassifications in the dataset)
c) Coastal refineries in importing countries which have the capabilities of shipping ports without having an explicit shipping port node near them (these cases are seen in South Asia and Iberia)

Of the above, categories a) and b) do not contribute to the goal of tracking supply chain pathways. In order to capture reasonable connectivity for cases in category c), we create artificial terminal locations coinciding with the refinery locations such that the corresponding terminal-

refinery pairs are connected. Category c) can be seen typically in importing countries that do not have extensive pipeline coverage (examples include Spain, Portugal, Japan).

Supplementary Note 4: Blend estimation algorithm

We estimate the formation of blends from crude oil at the level of oil fields as a means to estimating blend carbon intensities and tracking barrels from sources to destinations as shown by the schematic below:

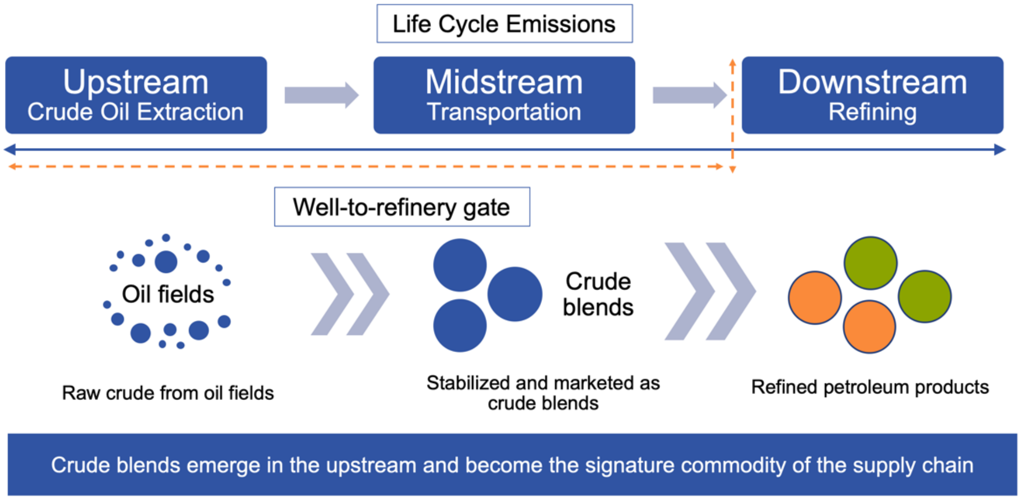

Supplementary Figure 4: Contextualizing crude blends within the supply chain

Formulation of the optimization problem

Recapping from the Methods section of the main paper, the configuration matrix Θ encodes the fraction of volumes from all oil fields contributing to make all crude blends in the given country i.e. Θ is a matrix of $F \times B$ dimensions where F is the total number of oil fields and B is the total number of crude blends such that the value corresponding to the ith row and jth column is the fraction of crude volume from field i that contributes to blend j.

$$\mathbf{\Theta} = \begin{bmatrix} \theta_{11} & \cdots & \theta_{1B} \\ \vdots & \ddots & \vdots \\ \theta_{F1} & \cdots & \theta_{FB} \end{bmatrix} \qquad (1)$$

The rest of the notation guiding the optimization problem is summarized below, with the dimensions included in parenthesis (see S.I. for more details)

$$\mathbf{V_F} = \text{Volume vector of oil fields } (F \times 1)$$

$$\mathbf{V_B} = \text{Volume vector of crude blends } (B \times 1)$$
$$\mathbf{A_F} = \text{API vector of oil fields } (F \times 1)$$
$$\mathbf{A_B} = \text{API vector of crude blends } (B \times 1)$$
$$\mathbf{D_F} = \text{Distance matrix of oil fields } (F \times F)$$
$$\mathbf{P_F} = \text{Connectivity matrix of oil fields } (F \times F)$$

The goal is to estimate the optimal $\boldsymbol{\Theta}$ i.e. $\boldsymbol{\Theta}^*$ given the following multi-objective cost function containing four sub-costs corresponding to distance (C_d), connectivity (C_c), volume (C_v) and API (C_a) (see S.I. for more details).

$$\boldsymbol{\Theta}^* = \text{argmin}_{\Theta} \left[\text{Cost}(C) = \sum_{i \epsilon \{d,c,v,a\}} w_i C_i \right] \qquad (2)$$

$$\text{such that } \forall\, i, \qquad w_i > 0 \quad \text{and} \left[\sum_{i \epsilon \{d,c,v,a\}} w_i \right] = 1; \qquad (3)$$

$$C_d = \left\| \left(\boldsymbol{\Theta}\boldsymbol{\Theta}^{\mathbf{T}} \right) \circ (\mathbf{D_F}) \right\|_1^1 \;;\; C_c = \left\| \left(\boldsymbol{\Theta}\boldsymbol{\Theta}^{\mathbf{T}} \right) \circ (\mathbf{P_F}) \right\|_1^1 \qquad (4)$$

$$C_v = \left\| \left(\mathbf{V_F^T} \boldsymbol{\Theta} \right)^{\mathbf{T}} - (\mathbf{V_B}) \right\|_1^1 \;;\; C_a = \left\| \left(\mathbf{A_F^T} \boldsymbol{\Theta} \right)^{\mathbf{T}} - (\mathbf{A_B}) \right\|_1^1 \qquad (5)$$

where $\circ$ is the Hadamard product and $||_1^1$ is the first norm

We summarize the interpretation of the sub-components in the Supplementary Table 2.

Supplementary Table 2: Components of blend estimation cost function

Sub-component	Interpretation	Formulation
C_d	To what extent do proximate oil fields make up the same blends	$\|\|(\boldsymbol{\Theta}\boldsymbol{\Theta}^{\mathbf{T}}) \circ (\mathbf{D_F})\|\|_1^1$
C_c	To what extent are oil fields contributing to the same blends connected in the supply chain network	$\|\|(\boldsymbol{\Theta}\boldsymbol{\Theta}^{\mathbf{T}}) \circ (\mathbf{P_F})\|\|_1^1$
C_v	How closely does the distribution of crude from oil fields manage to approximate the blend volumes	$\left\|\left(\mathbf{V_F^T}\boldsymbol{\Theta}\right)^{\mathbf{T}} - (\mathbf{V_B})\right\|_1^1$
C_a	How closely does the distribution of crude from oil fields manage to approximate the blend APIs	$\left\|\left(\mathbf{A_F^T}\boldsymbol{\Theta}\right)^{\mathbf{T}} - (\mathbf{A_B})\right\|_1^1$

C_d and C_c, respectively quantify the extent to which proximate and well-connected fields make up the same blends. $(\Theta\Theta^{\mathrm{T}})_{ij}$ indicates the co-blending of crude from field-*i* and field-*j*. Coupled with (D_F) and P_F through element-wise multiplication, it thus measures the cost associated with

distance and connectivity respectively. The formulation of C_v and C_a quantifies the difference between estimated and actual crude blend volume/API; $\left(\mathrm{V}_{\mathrm{F}}^{\mathrm{T}}\Theta\right)^{T}$ and $\left(\mathrm{A}_{\mathrm{F}}^{\mathrm{T}}\Theta\right)^{T}$ are the estimated crude blend volumes and APIs respectively. To ensure all cost terms are comparable, every sub-component is scaled using an estimate of its magnitude. This estimate is the sample mean of 100 sub-components computed by randomly sampling the configuration matrix subject to the constraint of every row summing to one (i.e. fractions of crude from an oil field allocated across all crude blends add to one).

Weights associated with the multi-objective cost function

The four weights associated with the cost function dictate the relative importance of the corresponding four sub-costs. The weights are set to 0.25 i.e. all are equal to assign sufficient importance to the sub-costs associated with volume, API, distance and connectivity. The weights are varied to quantify uncertainties shown in Figure 3 of the main paper. The choice of weights represents a direction for further improvement. With better and more data about the specifics of supply chains in different countries, there is significant room to better set the weights to reflect the physical realities of crude blending.

The initialization algorithm

The initialization algorithm is comprised of multiple modules executed in series which lead to a successive weighting of the configuration matrix.

A. Similarity scores based on entity names

Nomenclature of marketed crude blends is known to be region specific and occasionally, the same is observed in the case of oil fields. Furthermore, in cases where the blend originates from a specific cluster of fields which reside in the same basin, there are similarities in the way the blends and the fields are named. Taking advantage of this information, this module uses name similarity scores to bias the initialization of the configuration matrix. Similarity scores are calculated using the difflib library in Python which measures the degree to which two sequences are similar.

B. Genetic Algorithm

The configuration matrix has large dimensions which are variable (100s of oil fields and 10s of crude blends). This translates to a high number of parameters in the configuration matrix which makes the gradient descent susceptible to local minima traps[9]. This limitation hinders the gradient-descent from converging in a stable, robust manner. To mitigate this, a genetic algorithm, which is a non-gradient optimization method, is used in the initialization process[10]. The parameters of the genetic algorithm are the parent population size, offspring population size, fitness function, the chromosomes, and the number of iterations. Starting with the parent population, the offspring population is generated by crossing over chromosomes i.e. swapping the two halves of equisized field-to-blend arrays in the configuration matrix. Candidates in this

population are mutated by randomly varying one row in the matrix (corresponding to one field) and are selected for fitness by passing through the cost function. The choices for parameters and functions along with the behavior of the cost function are shown in Supplementary Table 3 and Supplementary Figure 5.

Supplementary Table 3: Hyperparameters of the genetic algorithm

Parameters and functions used in the genetic algorithm	Value
Parent population size	20
Fitness function	1 – Cost Function
Offspring population size	10
Chromosomes	Two equisized field-to-blend arrays
Iterations	5000

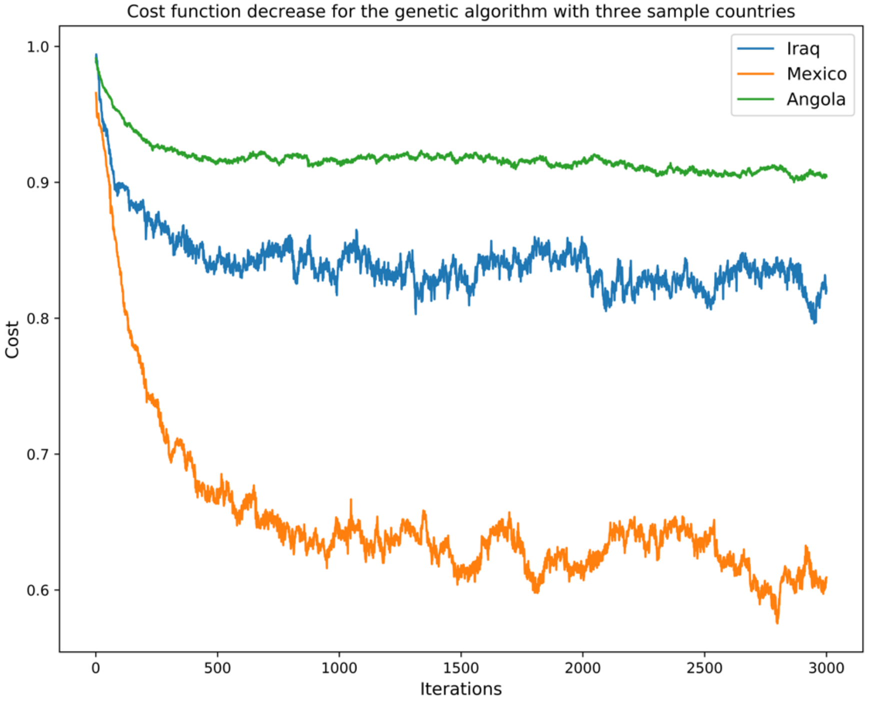

Supplementary Figure 5: Cost function decrease for the genetic algorithm for three sample countries focused on 0-3000 iterations

C. K-means clustering

Oil field clusters exhibit the property of being co-located in basins and other common geographical areas. This leads to certain crude blends being region specific and consequently being formed from the fields co-located in the respective regions. For a few cases, this common property reflects in the name similarity, but for the vast majority, it does not. This motivates a proximity-based module that groups oil fields based on their geolocations and forms blends from the emerging field clusters. K-means clustering is used given the nodular nature of the supply chain representation[11]. Oil field nodes represent data points to be clustered and crude blends represent the number of clusters to be formed. In order to emphasize the importance of fields who are major contributors to output, volume-weighted distancing is used to prioritize those with high production volumes. Sample counts of the field nodes and blends are shown in Supplementary Table 4.

Supplementary Table 4: **Examples of the k-means clustering setup in the initialization algorithm**

Country	Number of field nodes	Number of clusters = Number of blends
United States	1048	37
China	649	15
Saudi Arabia	35	6
Oman	176	1
Brazil	323	10

The cluster assignments are encoded into the initialization algorithm as follows:

$$\boldsymbol{\Theta}_{\mathbf{ij}} = \boldsymbol{\Theta}_{\mathbf{ij}} \times \boldsymbol{\epsilon} \quad if\ i \notin cluster\ j \qquad (6)$$
$$\boldsymbol{\Theta}_{\mathbf{ij}} = \boldsymbol{\Theta}_{\mathbf{ij}} \times \mathbf{M} \quad if\ i \in cluster\ j \qquad (7)$$
$$where\ \epsilon = 0.01\ and\ M = 100 \qquad (8)$$

Gradient descent using autodifferentiation

Gradient descent is performed starting with the initialized configuration matrix. Given the computational complexity of the cost function and non-linearity, gradient descent is implemented using autodifferentiation[9,12]. We use the deep learning framework PyTorch[13] for its computational graphs that facilitate the functionality of autodifferentiation. The graph allows for gradients to be computed at every step in the computation, which coupled with the chain rule of differentiation enables the computation of complicated gradients in a feasible manner. Gradient descent is implemented with the concept of momentum which does effective aver- aging over the

steps of descent. Momentum has been shown to make gradient descent more effective in training neural networks with large parameter spaces as shown by Qian et al[14]. Supplementary Table 5 summarizes the optimized hyperparameters that ensure stable convergence close to the local minima, Supplementary Figure 6 shows the pseudocode guiding the implementation of gradient descent and Supplementary Figure 7 shows the behavior of the cost function.

Supplementary Table 5: Hyperparameters of the gradient descent algorithm

Parameters in gradient descent	Value
Number of iterations	30000 – 50000 (depending on the size of the configuration matrix)
Step size	Variable (0.035 to 0.07) – indexed to iterations; steps size reduces as iterations increase
Momentum factor	0.9

function $gradient\_descent(\Theta_{init}, hyperparameters, cost\_function)$

$\Theta = \Theta_{init}$

$delta = \mathbf{0}$

```
// momentum factor - same dimensions as theta
```

for i in 1:niter

$C = cost\_function(\Theta)$

$delta = [\gamma \times delta] + [step \times \nabla C_{\Theta}]$

$\Theta = \Theta - delta$

```
// Gradient gradient descent with the momentum term.

// The backward method in PyTorch is used on the theta tensor inside every
 iteration of the cost function
```

end

Supplementary Figure 6: **Pseudocode for gradient descent with momentum and autodifferentiation**

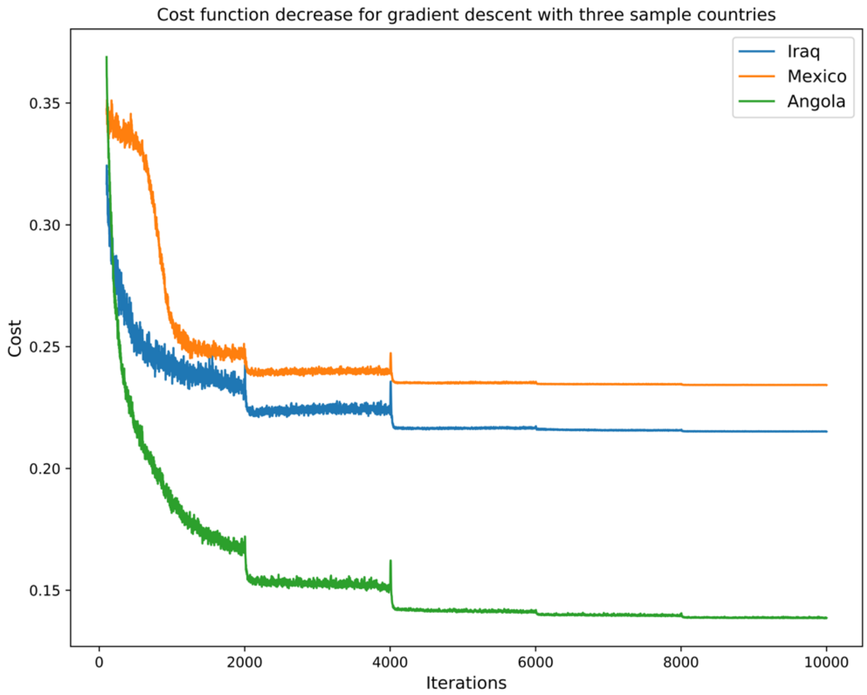

Supplementary Figure 7: **Cost function decrease for gradient descent for three sample countries focused on 100-10000 iterations**

Priority mode

For countries with a large number of oil fields and blends (eg: United States, Russian Federation, China), the aforementioned modules of the optimization are run on a priority set of fields. This priority set is generated by sorting fields in descending order of their volume and with a cutoff at 95 percent cumulative volume contribution. As a consequence, this picks the priority fields and leaves out the low volume contributors thus making the gradient descent more stable. The low contributors are allocated to blends based on the corresponding closest field from the priority set. The priority mode is activated only if the producing country has >100 unique oil fields. Specifically, if this criterion is satisfied, we sort oil fields in descending order of volume (higher volume first), then filter those fields which cumulatively represent 95 percent of the country's production volume (known as the priority set) followed by the other steps of the Blend Estimation Algorithm on the filtered set of fields and given set of blends. Lastly, for every residual low volume field, the blend assignment mirrors that of the closest field in the priority set.

Limitations

The blend estimation algorithm is implemented after filtering out condensates and natural gas

liquids (NGLs). These commodities typically have high API (low density) and interact with sister supply chains (eg: natural gas). Thus, this exclusion is a consequence of the available data that is limited to the oil supply chain.

Additionally, mass imbalances are observed in the key region of Texas affecting important blends like West Texas Intermediate, Eagle Ford. To mitigate these issues, the algorithm includes a module that selectively calibrates the initialization of the configuration matrix for the United States. This calibration is based on publicly available data about the likely origin oil fields of the major blends.

Sample cases of the Blend Estimation Algorithm

We show two views of the Blend Estimation Algorithm – the formation of a single blend and the formation of all blends in a given country. The left sub-figure in Supplementary Figure 8 shows the formation of the Kirkuk blend based on contributions from all oil fields in Iraq. Through the configuration matrix, the blend estimation algorithm captures several key insights about crude oil production in Iraq. Not only is the blend regionally constrained, but it is also linked to the Kirkuk region i.e. Northern part of Iraq sharing the same name. Similarly, by assigning fields to the predominant blends based on the configuration matrix, the algorithm captures those insights for other blends such as Basrah Light as shown in the right sub-figure.

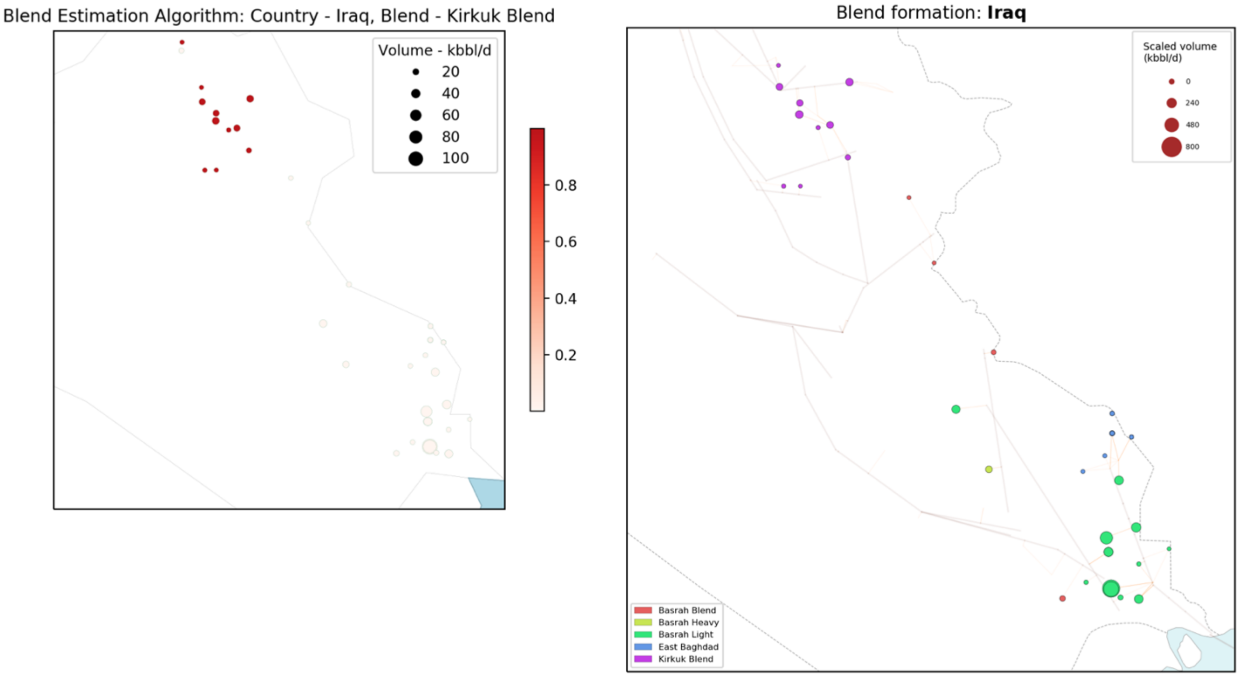

Supplementary Figure 8: Sample case study illustrating the blend estimation algorithm

Supplementary Note 5: Tracking crude from oil fields to refineries

With the optimal configuration matrix, oil fields are mapped to blends, which in turn are mapped to refineries through data sources mentioned in Supplementary Note 1. To estimate the specific supply chain routes for a given blend, we assume that all constituent fields supply crude to all destination refineries with the base production volume of the field weighted by the contribution factor of that field to the chosen blend and the fraction of the total blend volume consumed by the given refinery. We then find the shortest paths in the network starting from field nodes and ending at refinery nodes (shortest paths are computed using "real distances" i.e. length of pipeline segments and actual shipping distances as opposed to straight line or great circle distances). The assigned volumes are consolidated along all these paths and serve as the input for the midstream emission estimators. For cases where paths do not exist, we use source country to destination country volume-weighted averages for both pipeline and shipping emissions. Approximately ~70% of the total crude volume is captured by paths in the network and for the remaining ~30%, the choice of this default ensures that the emissions are governed by the transportation patterns between the specific source and destination countries.

Supplementary Note 6: Estimation of upstream emissions

The upstream carbon intensities for crude blends are derived from the blend configuration matrix. For a given blend, the upstream carbon intensity is the volume-weighted sum of the contributing fields' carbon intensities pre-multiplied by the configuration matrix as shown below:

$$\mathrm{Blend}_{j_{CI}} = \sum_{i=0}^{N_F} \Theta_{\mathrm{ij}} \times \mathrm{V_i} \times \mathrm{F_{i_{CI}}} \qquad (9)$$

where

Θ_{ij} is the contribution of field i in blend j,

V_i is the volume of field i and

$F_{i_{CI}}$ is the carbon intensity of field i

The upstream CI for global crude blends with volume greater than 100,000 bbl/day is shown in Supplementary Table 6.

Supplementary Table 6: Upstream CI for global crude blends with volume greater than 100,000 bbl/day

Producing Country	Blend	Upstream CI (gCO2eq/MJ)	Upstream CI (kgCO2eq/bbl)
Russian Federation	Domestic Oil Russia-4	9.33	50.92
Russian Federation	Domestic Oil Russia-1	9.42	52.04
Russian Federation	Siberian Light	8.76	47.99
Russian Federation	ESPO	9.27	51.40
Russian Federation	Sokol	8.39	46.07
Russian Federation	Domestic Oil Russia-3	7.86	44.63
Russian Federation	Urals-1	8.32	46.84
Russian Federation	Urals-2	8.16	45.95
Russian Federation	Domestic Oil Russia-2	9.45	52.79
Russian Federation	Urals-3	8.21	46.21
Colombia	Castilla	6.09	35.91
Colombia	Vasconia	7.55	43.31
Turkmenistan	Domestic Oil Turkmenistan	14.22	78.71
United Kingdom	Forties Blend	3.36	18.53
United Kingdom	Brent Blend	4.85	26.84
Egypt	Ras Gharib	9.78	56.60
Egypt	Suez Blend	8.32	46.30
Egypt	Western Desert Blend	9.40	51.51
Egypt	Qarun	9.20	50.96
Canada	Midale	7.57	42.45
Canada	Hibernia	5.77	31.99
Canada	Western Canadian Select	8.62	50.41
Canada	Other Oil Canada-1	8.71	47.01
Canada	Oil Sands Synthetic-1	25.75	144.52
Canada	Other Oil Canada-2	13.30	72.59
Canada	Cold Lake Blend	8.07	47.24
Canada	Suncor Synthetic H	10.18	60.24
Canada	Light Sour Blend	19.08	104.66
Algeria	Zarzaitine	14.46	77.77

Algeria	Saharan Blend	18.35	97.62
Azerbaijan	Domestic Oil Azerbaijan	3.72	20.56
Azerbaijan	Azeri Ligth-2	2.31	12.79
Azerbaijan	Azeri Ligth-1	2.31	12.79
Kuwait	Kuwait Export	2.33	13.04
Angola	Nemba Blend	9.37	50.79
Angola	Girassol	8.87	50.08
Angola	Dalia Blend	7.79	45.03
Angola	Cabinda Blend	9.54	53.30
Angola	Kuito Blend	8.48	49.40
Kazakhstan	CPC	10.76	57.59
Kazakhstan	Other Oil Kazakhstan-2	9.75	52.71
Kazakhstan	Other Oil Kazakhstan-1	9.52	52.69
United Arab Emirates	Upper Zakum	2.42	13.48
United Arab Emirates	Murban	5.23	28.28
United Arab Emirates	Das	4.28	23.47
Malaysia	Kikeh	11.89	65.86
Malaysia	Labuan	12.66	70.70
Libya	El Sharara	9.08	48.76
Brunei Darussalam	Champion	5.04	29.13
Argentina	Escalante	6.54	38.07
Argentina	Canadon Seco	5.30	30.54
Argentina	Domestic Oil Argentina-1	8.28	46.36
Argentina	Domestic Oil Argentina-2	9.44	51.84
Thailand	Domestic Oil Thailand	5.41	30.26
United States	Other Oil USA-3	7.93	44.29
United States	Light Louisiana Sweet	9.05	49.70
United States	Eagle Ford	8.51	45.37
United States	Southern Green Canyon Blend	5.63	31.59
United States	West Texas Intermediate	9.81	52.95
United States	Heavy Louisiana Sweet	9.60	53.50
United States	Alaska North Slope	7.70	43.62
United States	Wyoming Sweet	13.61	74.57

United States	Other Oil USA-2	8.58	46.33
United States	Mars Blend Deepwater	7.07	40.08
United States	Bakken	10.85	58.62
United States	Poseidon Blend Deepwater	7.19	40.64
United States	West Texas Sour	8.76	48.53
United States	Other Oil USA-1	31.14	185.64
Norway	Gullfaks Blend	5.72	31.30
Norway	Statfjord Blend	3.53	19.33
Norway	Ekofisk	2.08	11.38
Norway	Troll Blend	3.17	17.57
India	Domestic Oil India-2	5.41	30.86
India	Domestic Oil India-1	7.10	38.78
Mexico	Isthmus	8.52	47.26
Mexico	Maya	6.38	36.79
Mexico	Olmeca	9.39	51.02
Iraq	Basrah Heavy	7.95	45.51
Iraq	Basrah Light	12.56	71.61
Iraq	Kirkuk Blend	12.86	71.27
Venezuela	Merey	19.87	116.21
Venezuela	Mesa-30	15.46	86.86
Venezuela	Other Oil Venezuela-2	10.46	60.27
Venezuela	Other Oil Venezuela-3	14.26	83.78
Venezuela	Other Oil Venezuela-1	13.90	83.11
Venezuela	Tia Juana Pesado	19.36	116.89
Indonesia	Duri	31.61	184.17
Indonesia	Minas	21.80	120.20
Denmark	DUC	5.68	31.69
Oman	Oman Blend	9.20	51.47
Iran	Iran Light	16.87	94.09
Iran	Iran Heavy	12.34	69.17
Iran	Domestic Oil Iran-2	28.94	161.45
Iran	Domestic Oil Iran-1	7.49	41.53
Iran	Domestic Oil Iran-3	9.56	55.71

Congo	Djeno	10.80	61.54
Nigeria	Escravos	16.29	91.17
Nigeria	Brass River	14.00	76.04
Nigeria	Qua Iboe	10.87	60.20
Nigeria	Bonny Light	12.50	69.36
Nigeria	Yoho	12.65	68.42
Nigeria	Agbami-Ekoli	7.12	38.02
Nigeria	Forcados	12.92	72.70
Vietnam	Bach Ho	7.26	39.28
China	Peng Lai	5.21	30.35
China	Other Oil China-1	5.38	30.69
China	Domestic Oil China-1	4.45	24.54
China	Domestic Oil China-2	5.53	30.47
China	Shengli	6.06	34.65
China	Daqing	5.16	28.70
China	Other Oil China-2	3.73	21.73
China	Jilin	5.14	28.59
Saudi Arabia	Arab Light	1.45	8.02
Saudi Arabia	Arab Heavy	0.60	3.39
Saudi Arabia	Arab Medium	1.03	5.78
Saudi Arabia	Arab Extra Light	1.88	10.28
Saudi Arabia	Arab Super Light	2.54	13.19
Ecuador	Oriente	6.64	37.36
Qatar	Qatar Marine	6.00	33.47
Qatar	Qatar Land Blend	8.20	45.79
Qatar	Al-Shaheen	6.35	36.02
Brazil	Marlim	3.76	22.12
Brazil	Domestic Oil Offshore Brazil	3.57	20.43
Brazil	Roncador 28	5.07	28.59
Brazil	Lula	5.79	32.64

The uncertainties in upstream carbon intensity are quantified by varying the weights in the blend estimation algorithm. Specifically, the four weights are varied from [0 to 0.7] such that the weights sum to 1. This leads to a distribution of configuration matrices which in turn leads to a distribution of carbon intensities.

Supplementary Note 7: Estimation of midstream emissions

As mentioned in the main paper, pipeline emissions are estimated using COPTEM[15]. Specifically, after tracking crude oil from source fields to destination refineries globally, COPTEM is used on relevant pipeline segments found in these global pathways.

Data about pipeline geometries (diameter, length), properties of crude oil and geophysical features like ambient temperature and elevation are procured from sources mentioned in Supplementary Note 1. For each segment, the elevation change is calculated as the difference between elevation of the end point and the starting point. Offshore pipelines are assumed to have zero elevation. All crudes transported through the same pipeline are aggregated together (i.e., flow rate sum-up) for COPTEM runs and they share the same calculated GHG emissions intensity over the distance of this pipeline.

Since COPTEM computes energy requirement for pipeline transport based on the friction losses, which in turn are proportional to the length, all things equal, the C.I. estimates are directly proportional to length[15].

For pipelines with an outside diameter greater than 23 inches, a thickness of 0.5 inch is assumed; for pipelines with an outside diameter less than 23 inches, a thickness of 0.375 inch is assumed. Crude velocity is calculated from flow rate, pipeline outside diameter, and pipeline thickness. An upper bound of 4.6 m/s is adopted for velocity. Any segment that has a flow rate less than 500 bbl/d is assumed to have zero GHG emissions. For pipelines with unknown outside diameter, a velocity of 2.09 m/s (calculated volume weighted average flow velocity across the globe) and a thickness of 0.375 inch is assumed to calculate the outside diameter (a lower bound of 4-inch outside diameter is adopted). In addition, COPTEM default pipeline roughness factor 0.00015 CS-inch is used. Coupled with the API density data, crude viscosity is calculated using an improved temperature-viscosity correlation for crude oil systems (an upper bound of 350 cSt is set for viscosity). Ambient temperature data is coupled with crude inlet temperature which is assumed to be the COPTEM default of 38°C. Country-wise electricity grid emissions factor for 2017 is purchased from IEA[16].

In continuation to the description of the shipping emissions in the main paper, we present relevant additional details here. The raw AIS data is acquired from S&P Global and the ship parameters from the World Register of Ship (WRS) database. The IMO number, a unique seven-digit identifier for a vessel is used to extract AIS records of the identified crude oil tankers. To

translate tanker emissions from an absolute level ($kgCO_2eq$) to a metric of carbon intensity (i.e. per barrel - $kgCO_2eq$/barrel), we use the following tanker categories[17,18]:

Supplementary Table 7: Crude tanker types with DWT values and barrel capacities

Type	DWT (deadweight tonnage)	Barrel capacity
Small tanker	< 5000	19000
	5000 – 10000	48000
Handy	10000 – 20000	106000
	20000 – 30000	201000
	30000 – 40000	254000
	40000 – 60000	320000
Panamax	60000 – 80000	482000
Aframax	80000 – 120000	702000
Suezmax	120000 – 200000	1011000
VLCC	200000 – 320000	2040000
ULCC	> 320000	4000000

We then use the identified tankers in conjunction with the tracked crude pathways in the supply chain to map tankers to specific shipping routes between terminals. We then estimate emissions as described in the main paper using the following emission factors.

Supplementary Table 8: Emission factors used in the estimation of shipping emissions

Engine Type	CO_2 Emission Factor (g/kW-hr)	
	Heavy Fuel Oil (HFO)	Marine Gasoil (MGO) / Marine Diesel Oil (MDO)
Slow Speed Diesel (<130rpm)	620	589
Medium Speed Diesel	683	649

Gas Turbine	970	922
Auxiliary Engines	683	649
Auxiliary Boilers	970	922

The midstream CI for global crude blends with volume greater than 100,000 bbl/day is shown in Supplementary Table 9 below:

Supplementary Table 9: Midstream CI for global crude blends with volume greater than 100,000 bbl/day

Producing Country	Blend	Midstream CI (gCO2eq/MJ)	Midstream CI (kgCO2eq/bbl)
Russian Federation	Domestic Oil Russia-4	0.68	3.73
Russian Federation	Domestic Oil Russia-1	0.40	2.22
Russian Federation	Siberian Light	0.65	3.58
Russian Federation	ESPO	0.77	4.29
Russian Federation	Sokol	1.13	6.20
Russian Federation	Domestic Oil Russia-3	0.70	3.98
Russian Federation	Urals-1	0.60	3.38
Russian Federation	Urals-2	0.54	3.01
Russian Federation	Domestic Oil Russia-2	0.45	2.54
Russian Federation	Urals-3	0.89	5.01
Colombia	Castilla	1.48	8.70
Colombia	Vasconia	1.27	7.29
Turkmenistan	Domestic Oil Turkmenistan	0.04	0.22
United Kingdom	Forties Blend	0.28	1.55
United Kingdom	Brent Blend	0.57	3.13
Egypt	Ras Gharib	0.53	3.06
Egypt	Suez Blend	0.12	0.69
Egypt	Western Desert Blend	0.21	1.16
Egypt	Qarun	0.25	1.39
Canada	Midale	1.89	10.60
Canada	Hibernia	1.49	8.25
Canada	Western Canadian Select	2.15	12.57
Canada	Other Oil Canada-1	1.47	7.92
Canada	Oil Sands Synthetic-1	1.72	9.67

Canada	Other Oil Canada-2	1.75	9.58
Canada	Cold Lake Blend	2.16	12.66
Canada	Suncor Synthetic H	1.82	10.75
Canada	Light Sour Blend	1.87	10.25
Algeria	Zarzaitine	0.57	3.07
Algeria	Saharan Blend	0.42	2.24
Azerbaijan	Domestic Oil Azerbaijan	0.11	0.63
Azerbaijan	Azeri Ligth-2	1.17	6.45
Azerbaijan	Azeri Ligth-1	1.16	6.40
Kuwait	Kuwait Export	0.63	3.51
Angola	Nemba Blend	0.92	4.98
Angola	Girassol	1.20	6.79
Angola	Dalia Blend	1.08	6.24
Angola	Cabinda Blend	1.21	6.77
Angola	Kuito Blend	0.93	5.40
Kazakhstan	CPC	1.11	5.95
Kazakhstan	Other Oil Kazakhstan-2	2.59	13.98
Kazakhstan	Other Oil Kazakhstan-1	1.94	10.74
United Arab Emirates	Upper Zakum	0.58	3.22
United Arab Emirates	Murban	0.48	2.61
United Arab Emirates	Das	0.78	4.27
Malaysia	Kikeh	0.66	3.68
Malaysia	Labuan	0.84	4.69
Libya	El Sharara	0.61	3.27
Brunei Darussalam	Champion	0.67	3.85
Argentina	Escalante	0.68	3.93
Argentina	Canadon Seco	0.68	3.93
Argentina	Domestic Oil Argentina-1	0.11	0.61
Argentina	Domestic Oil Argentina-2	0.55	3.02
Thailand	Domestic Oil Thailand	0.11	0.61
United States	Other Oil USA-3	1.66	9.26
United States	Light Louisiana Sweet	1.33	7.32
United States	Eagle Ford	0.97	5.17
United States	Southern Green Canyon Blend	1.59	8.92
United States	West Texas Intermediate	1.93	10.44
United States	Heavy Louisiana Sweet	1.19	6.64

United States	Alaska North Slope	1.71	9.66
United States	Wyoming Sweet	0.80	4.40
United States	Other Oil USA-2	0.84	4.51
United States	Mars Blend Deepwater	1.71	9.67
United States	Bakken	3.10	16.73
United States	Poseidon Blend Deepwater	1.58	8.96
United States	West Texas Sour	2.93	16.25
United States	Other Oil USA-1	1.34	8.01
Norway	Gullfaks Blend	0.21	1.13
Norway	Statfjord Blend	0.26	1.43
Norway	Ekofisk	0.27	1.50
Norway	Troll Blend	0.06	0.32
India	Domestic Oil India-2	0.38	2.19
India	Domestic Oil India-1	0.26	1.43
Mexico	Isthmus	1.90	10.53
Mexico	Maya	1.19	6.84
Mexico	Olmeca	1.32	7.16
Iraq	Basrah Heavy	2.13	12.20
Iraq	Basrah Light	1.33	7.56
Iraq	Kirkuk Blend	0.56	3.10
Venezuela	Merey	1.79	10.46
Venezuela	Mesa-30	0.39	2.18
Venezuela	Other Oil Venezuela-2	0.40	2.30
Venezuela	Other Oil Venezuela-3	0.65	3.85
Venezuela	Other Oil Venezuela-1	0.71	4.23
Venezuela	Tia Juana Pesado	0.48	2.89
Indonesia	Duri	0.40	2.35
Indonesia	Minas	0.14	0.78
Denmark	DUC	0.25	1.41
Oman	Oman Blend	0.92	5.18
Iran	Iran Light	0.37	2.06
Iran	Iran Heavy	0.41	2.32
Iran	Domestic Oil Iran-2	0.43	2.39
Iran	Domestic Oil Iran-1	0.59	3.25
Iran	Domestic Oil Iran-3	0.59	3.42
Congo	Djeno	1.02	5.80

Nigeria	Escravos	0.71	4.00
Nigeria	Brass River	0.88	4.78
Nigeria	Qua Iboe	1.01	5.59
Nigeria	Bonny Light	1.06	5.87
Nigeria	Yoho	0.73	3.95
Nigeria	Agbami-Ekoli	0.99	5.30
Nigeria	Forcados	0.79	4.45
Vietnam	Bach Ho	0.49	2.67
China	Peng Lai	0.37	2.16
China	Other Oil China-1	0.26	1.51
China	Domestic Oil China-1	0.81	4.47
China	Domestic Oil China-2	0.72	3.99
China	Shengli	0.38	2.20
China	Daqing	0.34	1.90
China	Other Oil China-2	0.55	3.22
China	Jilin	0.52	2.89
Saudi Arabia	Arab Light	0.91	5.05
Saudi Arabia	Arab Heavy	0.80	4.54
Saudi Arabia	Arab Medium	1.29	7.21
Saudi Arabia	Arab Extra Light	0.80	4.37
Saudi Arabia	Arab Super Light	0.89	4.61
Ecuador	Oriente	1.33	7.48
Qatar	Qatar Marine	0.96	5.35
Qatar	Qatar Land Blend	0.72	4.01
Qatar	Al-Shaheen	0.60	3.41
Brazil	Marlim	0.71	4.20
Brazil	Domestic Oil Offshore Brazil	0.58	3.33
Brazil	Roncador 28	1.39	7.84
Brazil	Lula	0.66	3.73

Supplementary Note 8: Literature references for midstream emissions

The global volume-weighted midstream CI from our analysis is 5.37 kg-CO_2eq/bbl. This compares to the 6.0 kg-CO_2eq/bbl as reported by the IEA[19] (we note that the IEA analysis uses

2018 data).

The global volume-weighted average for shipping C.I. from our analysis is 2.61 kg-CO_2eq/bbl. This estimate compares to ~2.1 kg-CO_2eq/bbl from the analysis by Suzanne et al. that aggregates emissions at the trade-lane level[20] (note that the C.I. has been estimated from figure 2 of the main paper with a 159 liter per barrel conversion). Our C.I. estimate translates to 70 million tonnes in annual emissions for the ~73 million-barrels/day crude volume (note that we exclude NGLs and condensates). As part of the International Council of Clean Transportation's assessment, Olmer et al. attribute ~120 million tonnes in annual emissions to overall oil tanker activity[21]. Assuming an apportionment using the ratio of crude transport emissions and product transport emissions from the IEA estimates (ratio of 2 given crude transport emissions of 6.0 kg-CO_2eq/bbl and product transport emissions of 3.0 kg-CO_2eq/bbl)[19], this corresponds to maritime emissions of ~80 million tonnes from crude transport.

Supplementary Note 9: Contextualizing decarbonization based on crude CI differentiation

As indicated in the main paper, our analysis provides a granular estimate of "well to refinery entrance emissions" with a global volume weighted average of 50.46 kg-CO_2eq/bbl. The IEA's estimate for which is 57.23 kg-CO_2eq/bbl (based on 2018 data), which translates to total emissions of 1.9 Gt/year. Adding in refining and product transport emissions, the total emissions amount to 3.1 Gt/year[19]. Emissions of the overall sector (which include combustion) stand at ~12 Gt/year[22].

Thus, progression towards global climate goals in the context of the oil not only entails inter-sectoral decarbonization (e.g. evolving global energy mix), but also effective intra-sectoral decarbonization to get to those targets i.e. a supply trajectory optimized for emissions. Specifically, the latter entails two modalities – process-oriented interventions (e.g. reduced flaring, carbon capture) and market-oriented measures (e.g. incentives and policy for crude differentiation based on CI). Our analysis aims to create the fact-base to inform the latter and it is worth noting that both these modalities would operate together. In other words, emissions reductions through CI-based crude differentiation can be realized in addition to the process-oriented interventions.

As discussed in the main paper, the emission reduction opportunity of CI-based crude differentiation is 1.5 – 6.1 Gt up to 2050. Specifically, the size of the opportunity for the IEA Sustainable Development Scenario is ~4.5 Gt. This corresponds to a CI trajectory from our estimate of the current global volume weighted average of 50.46 kg-CO_2eq/bbl to 28.66 kg-CO_2eq/bbl if the supply curve would be optimized for CI. This translates to a 43.2% decrease in

CI over 35 years. We put this into context with the emission reduction opportunity from process-oriented decarbonization options – the IEA estimates a 43.7% CI reduction potential through a combination of a variety of measures including reducing venting and flaring, methane reduction, use of renewables in operations, carbon capture etc. under the Sustainable Development Scenario from 2018 to 2030[23] i.e. over 12 years. Thus, market-based solutions which would incentivize CI-based crude differentiation, could be roughly a third as impactful as process-oriented interventions (comparable reduction potential over 35 years v/s 12 years) at a significantly lower capital intensity[24].

Supplementary Note 10: Relevant details on estimation of field-level upstream emissions

One of the major developments in this study is the use of OPGEE's most recent and detailed version (v3.0) to estimate the oil production carbon intensities worldwide at field level[1–3]. This is a significant advancement from the earlier OPGEE version (2.0b) used by Masnadi et al[1]. The upstream model improvements include, but are not limited to, accounting of venting and fugitive emissions and key processes related to gas systems. Further explanation of these enhancements is provided below:

The updated components related to fugitives in the new OPGEE model harnesses the most comprehensive publicly available database of component-level activity and emissions measurements, encompassing six studies and approximately 3,200 measurements[25]. Comparative studies using the new fugitives' model have consistently highlighted several critical empirical features.

The updated model (1) generates higher total emissions than the EPA GHGI, with the most substantial discrepancies arising from liquid hydrocarbon storage tanks and equipment leakage; (2) exhibits more substantial dependence on-site gas productivity for loss fraction; and (3) features pronounced "heavy-tailed" emissions distributions, which rely on large emitters to account for a significant fraction of emissions[25].

In addition to fugitives, the second key enhancement is related to gas gathering systems. Gas gathering systems (GGS) serve as crucial components within the midstream sector of the oil and gas industry, providing an essential interface between production and utilization. They are responsible for the collection, segregation, and often, the processing of natural gas following its extraction before it reaches residential or commercial establishments[26].

GGS primarily comprises an intricate network of small-diameter pipelines that transport the gas from the wellhead to more extensive gathering lines, which, in turn, direct it to the processing

plant. Typically, these gathering lines are smaller than eight inches in diameter and operate at low pressures, predominantly in rural production areas[27]. In the context of OPGEE, the GGS is a binary variable. This variable significantly influences the fractions of storage gas recovery and gas flaring. Specifically, upon activation of the GGS, the system sets the gas recovery fraction at 90% and the gas flare fraction at 10%, a significant deviation from the default values of 6% for gas recovery and 43% for gas flaring. While the default values are tailored to U.S. crude oil production, the activation of GGS is when considering other regions.

A comprehensive study outlining these latest enhancements in the Oil Production Greenhouse Gas Emissions Estimator (OPGEE) model is currently underway and will be published in the near future.

Additionally, we present comparative references to OPGEE from literature below. Supplementary Table 10 compares various emission estimation methods used in the upstream oil and gas sector. Among these methods, OPGEE showcases significant advantages over its counterparts. Comparing OPGEE with other bottom-up models, two major strengths are immediately discernible. Firstly, OPGEE is an open-source model that promotes transparency and allows for public validation and verification, a feature often lacking in other models. Secondly, the breadth of production methods that OPGEE can model surpasses other bottom-up approaches. It encompasses a diverse range of methods, such as gas lifting and flooding, and includes a broader selection of processing units.

When compared with the U.S. Environmental Protection Agency's Greenhouse Gas Reporting Program (GHGRP), OPGEE's capability extends beyond the confines of US fields, rendering it a more versatile and globally applicable model. In relation to top-down methodologies, like aerial surveys and satellite monitoring, OPGEE has the unique advantage of computing CO_2 equivalents, which include both CO_2 and CH_4 emissions. Existing top-down techniques are predominantly limited to measuring CH_4 emissions. Furthermore, the relative error in these top-down approaches under a single-blind test can be extensive, ranging from -100% to 100%[28], demonstrating the superior precision of OPGEE.

Compared to commercial entities like Project Canary[29], which require users to install sensors on equipment and conduct measurements, OPGEE represents a more cost-effective and efficient solution. Therefore, for comprehensive, accessible, and accurate estimation of greenhouse gas emissions in the oil and gas sector, OPGEE emerges as a distinctly powerful tool.

Supplementary Table 10: Comparative Review of Emission Estimation Methods in the Upstream Oil and Gas Sector

Method Name	Method Type	Emissions Type	System Boundary	Modeling Range	Open-source
OPGEE[1,3]	Bottom-up	CO_2e	well-to-refinery	Global	Yes
FUNNELGHG-COO[30]	Bottom-up	CO_2e	Well-to-tank	Conventional oil	No
FUNNELGHG OS[30]	Bottom-up	CO_2e	well-to-tank	Oil sands	No
GHGRP[31,32]	Direct Report	CO_2e	Well-to-refinery	U.S.	Yes
Aerial Survey[33]	Top-down	CH_4	N.A.	Global	No
Carbon Mapper Satellite[34]	Top-down	CH_4	N.A.	Global	Yes
Project Canary[29]	Direct Measurement	CH_4	N.A.	Global	No

Supplementary Note 11: Data gaps and future research

Natural gas gathering and boosting infrastructure (i.e., pipelines) can play a key role in oil and gas production emissions. These pipelines are most often operated via compressor stations at pressures of 100–2,000 kPa, which is lower than transmission pipelines but higher than distribution pipelines. Methane emissions can originate from three main sources, including underground pipeline leaking, above-ground auxiliary equipment leaking, and intentional venting (e.g., maintenance blowdown)[35,36]. To date, few studies have characterized emissions from such infrastructure using a top-down approach because the scale and complexity of gathering pipeline networks and the fact that many are underground and in difficult-to-access locations.

U.S. EPA[31] reported that in 2021, emissions from gathering and boosting infrastructure were almost on par with those from oil and gas production emissions on an absolute basis (both around 90 million metric tons CO_2e). A nation-wide gathering pipeline emissions factor of 0.19 Mg year^{-1} km^{-1} considering 710,000 km gathering pipeline was estimated by U.S. EPA[37]. Several other studies[38–40] focusing on ground-based surveys showed that gathering pipeline emissions are 0.75 Mg year^{-1} km^{-1} at the most. Yu et al.[26] conducted aerial-based campaigns in the Permian basin and reported that emissions factor estimates for gathering pipelines can range from 2.7 (+1.9/–1.8) Mg year^{-1} km^{-1} for Fall 2021 to 10.0 (+6.4/–6.2) Mg year^{-1} km^{-1} for Fall

2019. The discrepancy in estimates between the ground- and aerial-based studies may be due to area covered and sampling size. Therefore, the distance of gathering pipelines can be a crucial factor in estimating such fugitive emissions.

However, it is noteworthy that the vast majority of gathering and boosting emissions are associated with natural gas production and processing. In this study, we focus on oil producing fields with a cutoff gas-to-oil ratio at 10,000 scf/bbl, meaning that any fields producing more gas than this cutoff ratio are excluded. Therefore, the contribution of gathering and boosting emissions to the emissions factor for crude oil is much less than that for natural gas. In addition, information about global gathering and boosting pipelines is not available for estimating the associated emissions on a bottom-up basis. Nonetheless, OPGEE v3.0 does have a component-level fugitive emissions model that estimates methane emissions from storage tank and equipment leakage based on a comprehensive literature review of component-level activity and emissions measurements[25]. This may help cover part of the aforementioned gathering and boosting emissions but is less ideal than having bottom-up estimates using gathering and boosting pipeline infrastructure information (e.g., distance) and region-specific emissions factors.

The fugitive emissions model OPGEE v3.0 employs is constructed upon the most comprehensive public datasets available to date, encompassing component-level equipment counts and emissions[25]. However, these datasets are based solely on United States data. This specificity introduces certain limitations, given the differences between the upstream oil and gas operations in the United States and other regions worldwide.

In particular, the extrapolation of the US-centric data to a global scale may generate errors in estimating component-level loss rates, especially considering the variability and heterogeneity of operational practices, technologies, and regulatory standards across the globe. Such discrepancies highlight the potential value of integrating region-specific datasets and developing a globally representative and accurate model.

In addition, a secondary constraint within the model's design relates to the scope of emissions it can accurately predict. As depicted in Supplementary Figure 35 by Rutherford et al.[25], the model's predicted maximum well-site CH_4 emissions cap at 100 kg/h. However, evidence provided by Chen et al.[33] via a comprehensive aerial survey delineates a broader range for well-site CH_4 emissions, extending from 10 kg/h to as high as 10,000 kg/h.

Analysis of the probability density distribution indicates the peak of CH_4 emissions to be approximately around 100 kg/h. However, when evaluating the cumulative density distribution, emissions surpassing 100 kg/h account for a substantial 75% of total emissions. This substantial underestimation of emissions intensity highlights the necessity for model adjustments to encompass higher-end emissions values. Overall, these identified limitations point towards the

potential for developing more comprehensive and geographically representative models to accurately estimate emissions in the global upstream oil and gas sector.

Supplementary References

1. Masnadi, M. S. *et al.* Global carbon intensity of crude oil production. *Science* **361**, 851–853 (2018).
2. El-Houjeiri, H. M., Brandt, A. R. & Duffy, J. E. Open-source LCA tool for estimating greenhouse gas emissions from crude oil production using field characteristics. *Environmental Science & Technology* **47**, 5998–6006 (2013).
3. Long, W. & Brandt, A. R. OPGEE v3.0c. *GitHub repository* (2015).
4. GlobalData, Oil and Gas. *https://www.globaldata.com/industries-we-cover/oil-gas/*.
5. Kpler - Leading Commodity Data and Analytics Solution. *https://www.kpler.com/*.
6. IHS Markit, Shipping. *https://ihsmarkit.com/products/ship-and-port-data.html*.
7. Wan, Zhengming, Hook, Simon, & Hulley, Glynn. *MOD11C3 MODIS/Terra Land Surface Temperature/Emissivity Monthly L3 Global 0.05Deg CMG V006*. (NASA EOSDIS Land Processes DAAC, 2015). doi:10.5067/MODIS/MOD11C3.006.
8. de Ferranti, J. Digital elevation data with SRTM voids filled using accurate topographic mapping. *http://viewfinderpanoramas.org/dem3.html* (2021).
9. Ruder, S. An overview of gradient descent optimization algorithms. *arXiv preprint arXiv:1609.04747* (2016).
10. Vose, M. D. *The simple genetic algorithm: foundations and theory*. (MIT press, 1999).
11. Likas, A., Vlassis, N. & Verbeek, J. J. The global k-means clustering algorithm. *Pattern recognition* **36**, 451–461 (2003).
12. Paszke, A. *et al.* Automatic differentiation in pytorch. (2017).
13. Paszke, A. *et al.* Pytorch: An imperative style, high-performance deep learning library. *arXiv preprint arXiv:1912.01703* (2019).
14. Qian, N. On the momentum term in gradient descent learning algorithms. *Neural Networks* **12**, 145–151 (1999).
15. Choquette-Levy, N., Zhong, M., MacLean, H. & Bergerson, J. COPTEM: a model to investigate the factors driving crude oil pipeline transportation emissions. *Environmental Science & Technology* **52**, 337–345 (2018).
16. IEA Emission Factors, 2019.
17. Stopford, M. *Maritime Economics 3e*. (Routledge, 2008).
18. *Oil tanker sizes range from general purpose to ultra-large crude carriers on AFRA scale - Today in Energy - U.S. Energy Information Administration (EIA)*.
19. IEA, Spectrum of the well-to-tank emissions intensity of global oil production, 2018, Paris *https://www.iea.org/data-and-statistics/charts/spectrum-of-the-well-to-tank-emissions-intensity-of-global-oil-production-2018*.
20. Greene, S., Jia, H. & Rubio-Domingo, G. Well-to-tank carbon emissions from crude oil maritime transportation. *Transportation Research Part D: Transport and Environment* **88**, 102587 (2020).

21. Olmer, N., Comer, B., Roy, B., Mao, X. & Rutherford, D. Greenhouse Gas Emissions from Global Shipping, 2013–2015 Detailed Methodology. *International Council on Clean Transportation: Washington, DC, USA* 1–38 (2017).
22. Hannah Ritchie, M. R. & Rosado, P. CO_2 and Greenhouse Gas Emissions. *Our World in Data* (2020).
23. IEA, Changes in the average global emissions intensity of oil and natural gas operations in the Sustainable Development Scenario, 2018-2030. *https://www.iea.org/data-and-statistics/charts/changes-in-the-average-global-emissions-intensity-of-oil-and-natural-gas-operations-in-the-sustainable-development-scenario-2018-2030.*
24. IEA, Marginal abatement cost curve for oil- and gas-related methane emissions by mitigation measure, 2019 *https://www.iea.org/data-and-statistics/charts/marginal-abatement-cost-curve-for-oil-and-gas-related-methane-emissions-by-mitigation-measure-2019.*
25. Rutherford, J. S. *et al.* Closing the methane gap in US oil and natural gas production emissions inventories. *Nature Communications* **12**, 4715 (2021).
26. Yu, J. *et al.* Methane Emissions from Natural Gas Gathering Pipelines in the Permian Basin. *Environmental Science & Technology Letters* **9**, 969–974 (2022).
27. Tallant, J. *Gas gathering, measurement, and processing, 2023.*
28. Sherwin, E. D. *et al.* Single-blind validation of space-based point-source detection and quantification of onshore methane emissions. *Scientific Reports* **13**, 3836 (2023).
29. *Project Canary. Advanced continuous emissions monitoring (cem) guide*. https://www.projectcanary.com/.
30. Nimana, B., Canter, C. & Kumar, A. Energy consumption and greenhouse gas emissions in upgrading and refining of Canada's oil sands products. *Energy* **83**, 65–79 (2015).
31. U.S. EPA. *GHGRP Petroleum and Natural Gas Systems (2022)*. https://www.epa.gov/ghgreporting/ghgrp-petroleum-and-natural-gas-systems#subsector.
32. Statistics, C. Greenhouse gas reporting program. GHGRP 2016: Reported Data (2018).
33. Chen, Y. *et al.* Quantifying regional methane emissions in the New Mexico Permian Basin with a comprehensive aerial survey. *Environmental Science & Technology* **56**, 4317–4323 (2022).
34. Cusworth, D. H. *et al.* Strong methane point sources contribute a disproportionate fraction of total emissions across multiple basins in the United States. *Proceedings of the National Academy of Sciences* **119**, e2202338119 (2022).
35. Marchese, A. J. *et al.* Methane emissions from United States natural gas gathering and processing. *Environmental science & technology* **49**, 10718–10727 (2015).
36. Zimmerle, D. *et al.* Methane emissions from gathering compressor stations in the us. *Environmental Science & Technology* **54**, 7552–7561 (2020).
37. *U.S. EPA. Annex 3.6: Methodology for Estimating CH4, CO2, and N2O Emissions from Natural Gas Systems (2022)*. https://www.epa.gov/sites/default/files/2017-02/documents/3._6_natural_gas_systems_annex_2017-2-10_.pdf.
38. Zimmerle, D. J. *et al.* Gathering pipeline methane emissions in Fayetteville shale pipelines and scoping guidelines for future pipeline measurement campaigns. *Elem Sci Anth* **5**, 70 (2017).
39. Li, H. Z., Mundia-Howe, M., Reeder, M. D. & Pekney, N. J. Gathering pipeline methane emissions in utica shale using an unmanned aerial vehicle and ground-based mobile sampling. *Atmosphere* **11**, 716 (2020).

40. Li, H. Z., Mundia-Howe, M., Reeder, M. D. & Pekney, N. J. Constraining natural gas pipeline emissions in San Juan Basin using mobile sampling. *Science of the Total Environment* **748**, 142490 (2020).
