# Supplementary material for: Carbon intensity of global crude oil trading and market policy implications
Source: Nat Commun. 2023 Sep 25;14:5975. doi: 10.1038/s41467-023-41701-z (PMC10520038; doi:10.1038/s41467-023-41701-z)
Supplement: Supplementary file 3 — Reporting Summary [file 41467_2023_41701_MOESM3_ESM.pdf]

nature portfolio

Corresponding author(s): Raymond L. Speth

Last updated by author(s): Sep 3, 2023

Reporting Summary

Nature Portfolio wishes to improve the reproducibility of the work that we publish. This form provides structure for consistency and transparency in reporting. For further information on Nature Portfolio policies, see our Editorial Policies and the Editorial Policy Checklist.

Statistics

For all statistical analyses, confirm that the following items are present in the figure legend, table legend, main text, or Methods section.

n/a	Confirmed	
☒	☐	The exact sample size (*n*) for each experimental group/condition, given as a discrete number and unit of measurement
☒	☐	A statement on whether measurements were taken from distinct samples or whether the same sample was measured repeatedly
☒	☐	The statistical test(s) used AND whether they are one- or two-sided<br>*Only common tests should be described solely by name; describe more complex techniques in the Methods section.*
☒	☐	A description of all covariates tested
☒	☐	A description of any assumptions or corrections, such as tests of normality and adjustment for multiple comparisons
☐	☒	A full description of the statistical parameters including central tendency (e.g. means) or other basic estimates (e.g. regression coefficient) AND variation (e.g. standard deviation) or associated estimates of uncertainty (e.g. confidence intervals)
☒	☐	For null hypothesis testing, the test statistic (e.g. *F*, *t*, *r*) with confidence intervals, effect sizes, degrees of freedom and *P* value noted<br>*Give P values as exact values whenever suitable.*
☒	☐	For Bayesian analysis, information on the choice of priors and Markov chain Monte Carlo settings
☒	☐	For hierarchical and complex designs, identification of the appropriate level for tests and full reporting of outcomes
☒	☐	Estimates of effect sizes (e.g. Cohen's *d*, Pearson's *r*), indicating how they were calculated

Our web collection on statistics for biologists contains articles on many of the points above.

Software and code

Policy information about availability of computer code

Data collection	Datasets sourced from commercial and publicly available sources - upstream data from Wood Mackenzie (commercial), midstream data from GlobalData, Kpler and IHS Markit (commercial) and downstream data from Wood Mackenzie (commercial); geographical data from NASA MODIS Land/Ocean Surface Temperature and Emissivity dataset, 2000 Shuttle Radar Topography Mission and NASA SRTM (publicly available); refer to Supplementary Information for more details.
Data analysis	Analysis was performed in Python using: NetworkX 2.1 for network analysis of the supply chain, PyTorch (torch 1.3.0) for creating the computation graph to perform optimization, Scikit-learn for k-means clustering, and NumPy 1.15.4 and pandas 1.0.3 for data processing; The code is available at https://doi.org/10.5281/zenodo.6814485.

For manuscripts utilizing custom algorithms or software that are central to the research but not yet described in published literature, software must be made available to editors and reviewers. We strongly encourage code deposition in a community repository (e.g. GitHub). See the Nature Portfolio guidelines for submitting code & software for further information.

Data

Policy information about availability of data

All manuscripts must include a data availability statement. This statement should provide the following information, where applicable:

- Accession codes, unique identifiers, or web links for publicly available datasets
- A description of any restrictions on data availability
- For clinical datasets or third party data, please ensure that the statement adheres to our policy

The upstream CI data for global crude blends generated in this study are provided in the Supplementary Information, Note 6. The midstream CI data for global crude blends generated in this study are provided in the Supplementary Information, Note 7. The oil field, shipping terminal, pipeline, refinery, crude trade, and shipping route data that support the findings of this study are collectively available from Wood Mackenzie, GlobalData, Kpler, and S&P Global; commercial restrictions apply to the availability of these data, which were used under license for the current study, and so are not publicly available. The earth surface temperature data used in this study are available from NASA MODIS. The elevation data used in this study are available from the NASA Shuttle Radar Topography Mission. Source data are provided with this paper.

Human research participants

Policy information about studies involving human research participants and Sex and Gender in Research.

Reporting on sex and gender	Human research not applicable
Population characteristics	Human research not applicable
Recruitment	Human research not applicable
Ethics oversight	Human research not applicable

Note that full information on the approval of the study protocol must also be provided in the manuscript.

Field-specific reporting

Please select the one below that is the best fit for your research. If you are not sure, read the appropriate sections before making your selection.

☐ Life sciences ☐ Behavioural & social sciences ☒ Ecological, evolutionary & environmental sciences

For a reference copy of the document with all sections, see nature.com/documents/nr-reporting-summary-flat.pdf

Ecological, evolutionary & environmental sciences study design

All studies must disclose on these points even when the disclosure is negative.

Study description	High resolution estimation of the carbon intensity of crude oil trading using a well-to-refinery entrance life cycle assessment
Research sample	Global oil fields (10460 count, ~98% coverage by volume), all global crude blends (326 count, excluding NGLs and condensates) and all global refineries (746 count); all based on 2015 supply chain data
Sampling strategy	Sampling is not applicable to the analysis since the optimization approach is performed for all oil fields and crude blends
Data collection	No data collection beyond the commercial and public datasets used (refer to the "Data" section) above
Timing and spatial scale	Supply chain data used from 2015 with global coverage
Data exclusions	NGLs and condensates excluded to maintain scope to the oil supply chain without including low-density entities in the blend estimation process (i.e. associating production from oil fields with global marketed crude blends)
Reproducibility	30 iteration cycles performed for each country-specific optimization run in the blend estimation algorithm; multi-iteration approach was chosen to ensure stability and reproducibility of optimal solutions; 10 iteration cycles for all 6 parameter combinations (60 iterations in all) used to quantify uncertainties in optimal estimate (refer to Methods in Main Paper and S.I. for more details)
Randomization	Randomization not applicable since entities are not segmented to groups
Blinding	Blinding not applicable since no particular group-level observational assessment was performed

Did the study involve field work? ☐ Yes ☒ No

Reporting for specific materials, systems and methods

We require information from authors about some types of materials, experimental systems and methods used in many studies. Here, indicate whether each material, system or method listed is relevant to your study. If you are not sure if a list item applies to your research, read the appropriate section before selecting a response.

Materials & experimental systems

n/a	Involved in the study
☒	☐ Antibodies
☒	☐ Eukaryotic cell lines
☒	☐ Palaeontology and archaeology
☒	☐ Animals and other organisms
☒	☐ Clinical data
☒	☐ Dual use research of concern

Methods

n/a	Involved in the study
☒	☐ ChIP-seq
☒	☐ Flow cytometry
☒	☐ MRI-based neuroimaging

March 2021
